# Retraction: Study on the Effect of Berberine, Myoinositol, and Metformin in Women with Polycystic Ovary Syndrome: A Prospective Randomised Study

**DOI:** 10.7759/cureus.r259

**Published:** 2026-11-02

**Authors:** Neha Mishra, Ruchi Verma, Payal Jadaun

**Affiliations:** 1 Obstetrics and Gynaecology, Goverment Institute of Medical Sciences, Greater Noida, IND; 2 Obstetrics and Gynaecology, Veerangana Avantibai Lodhi Autonomous State Medical College, Etah, IND

This article has been retracted by the Editor-in-Chief due to errors in the reported data, including the sample size. The underlying data could not be verified. The corresponding author agrees with the decision to retract.

